# Evolution of size-selected Pt cluster catalysts on prototypical oxide supports

**DOI:** 10.1039/d6fd00002a

**Published:** 2026-03-11

**Authors:** Lorenz J. Falling, Maximilian Huber, Johanna Reich, Matthias Krinninger, Sebastian Kaiser, Markus Döblinger, Marian D. Rötzer, Maximilian Krause, Andrey Shavorskiy, Suyun Zhu, Ueli Heiz, Hendrik Bluhm, Friedrich Esch, Barbara A. J. Lechner

**Affiliations:** a Functional Nanomaterials Group & Catalysis Research Center, Department of Chemistry, TUM School of Natural Sciences, Technical University of Munich Lichtenbergstr. 4 85748 Garching Germany bajlechner@tum.de; b Chemical Sciences Division & Advanced Light Source, Lawrence Berkeley National Laboratory Berkeley California 94720 USA; c Chair of Physical Chemistry & Catalysis Research Center, Department of Chemistry, TUM School of Natural Sciences, Technical University of Munich Lichtenbergstr. 4 85748 Garching Germany; d Department of Chemistry & Center for NanoScience (CeNS), University of Munich (LMU) Butenandtstr. 11 81377 Munich Germany; e MAX IV Laboratory, Lund University Lund 221 00 Sweden; f Institute for Advanced Study, Technical University of Munich Lichtenbergstr. 4 85748 Garching Germany

## Abstract

The current quest for new pathways into sustainable, efficient and durable energy conversion technologies makes the used for a fundamental understanding of the atomic-scale phenomena underlying catalytic processes ever more pressing. In this context, characterizing catalyst particles *in situ* provides valuable information about the evolution of their composition, structure, oxidation state and charge state during an ongoing process. To disentangle the influence of individual parameters – temperature, pressure, gas composition, cluster size, as well as support acidity, redox state and defect density – it is crucial to control them precisely and separately in experiments. At the example of size-selected Pt_*n*_ clusters – *i.e.* sub-nm particles defined to the exact number of atoms – on flat oxide supports, we follow their rich evolution phenomena *via* (synchrotron-based) X-ray photoelectron spectroscopy (XPS) and scanning tunnelling microscopy (STM) during temperature ramps and in various gas environments. Here, we present our experience with these highly defined, yet complicated-to-create samples in ultra-high vacuum (UHV) and at mbar pressures. We discuss their stability during transport to synchrotrons and under reaction conditions on three prototypical oxide supports and show the various phenomena that can be disentangled. Pt_*n*_ clusters on the non-reducible silicon dioxide, SiO_2_, remain size-selected and show remarkable stability. Doping strongly influences the size-dependent binding energy shifts and changes the Pt response to oxidative and reaction conditions, which we attribute to different cluster geometries: p-type doping leads to wetting, enhanced sinter resistance and a diminished response to oxidative environments, compared to clusters on n-type doped samples. We compare the system with our previous findings of a similar change in dimensionality for Pt_20_ clusters on the reducible ceria, CeO_2_(111), support, induced by modulation of its O vacancy density. The Pt_*n*_/CeO_2_ system is particularly interesting for strategies to stabilize and redisperse Pt clusters dynamically. Finally, we study the evolution of Pt_*n*_ clusters on another reducible oxide support, magnetite, Fe_3_O_4_(001), in 0.1 mbar alternating redox conditions at RT and elevated temperature. In analogy to findings previously reported for Pt/TiO_2_(110), the clusters either become encapsulated by a thin oxide film *via* strong metal–support interaction (SMSI) or deeply buried in the magnetite. Overall, our approach of following the evolution of size-selected clusters on oxide supports leads to fundamental atomic-scale insights on nano-scale catalyst materials, on our path to sustainable, dynamic and self-repairing catalysts.

## Abstract 2

In this comparative study of size-selected Pt clusters on single crystalline oxide supports, we consider the effects of support doping and oxidation state, gas composition and pressure, temperature and cluster size using NAP-XPS and STM.

## Graphical abstract

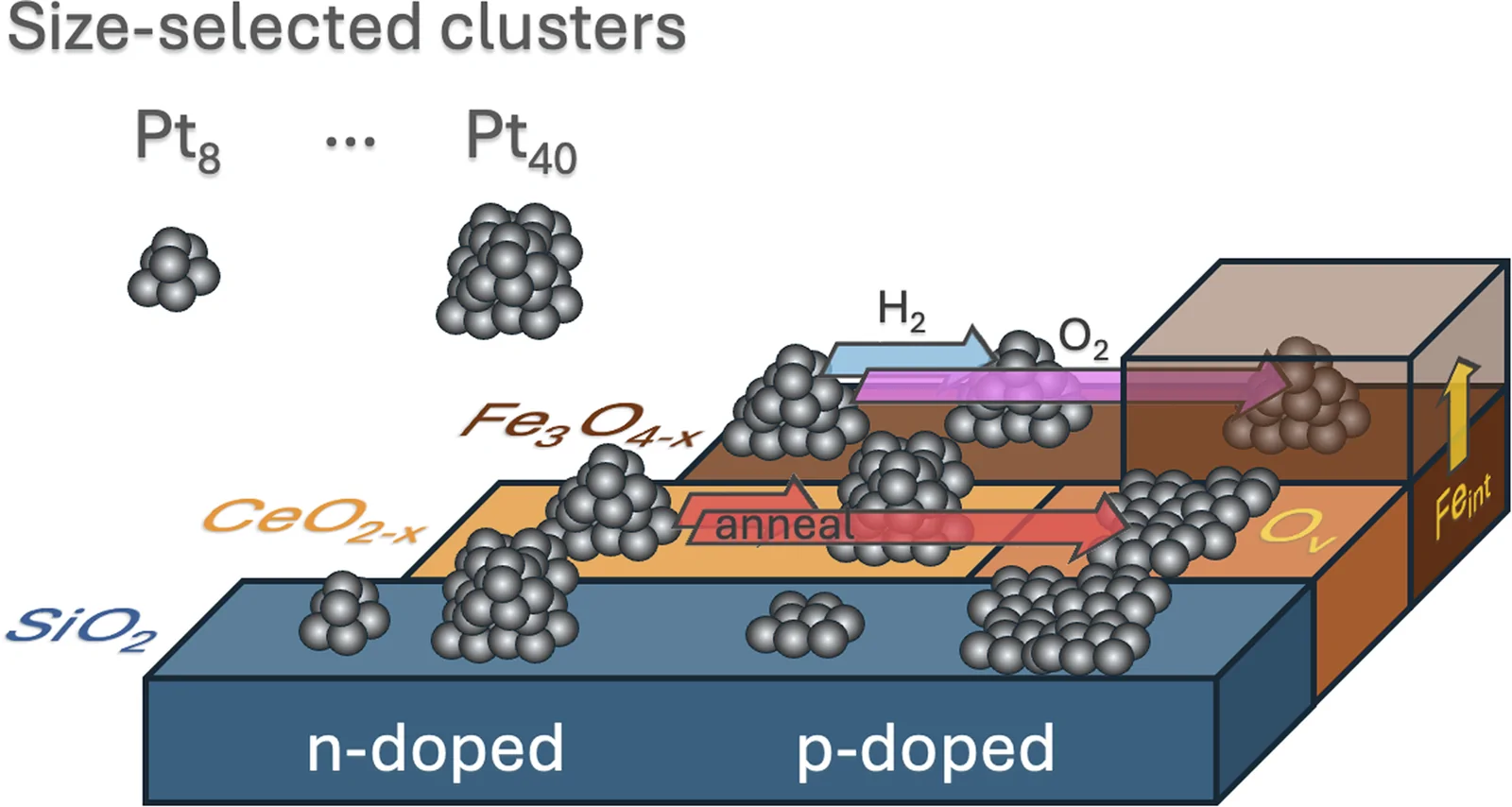

## Introduction

From fine chemical synthesis over exhaust control to electro- and photocatalytic energy conversion, most chemical processes rely on catalysts to enhance energy and material efficiency. Metal nanoparticles make up the majority of heterogeneous catalysts,^1^ as they have a high surface-to-volume ratio and are typically much more active than their bulk counterparts. Clusters – here defined as particles <1 nm in diameter – are even smaller and exhibit even more peculiar properties.^2,3^ Metal clusters like Pt or Pd, for example, open up a band gap.^4–6^ Clusters are in the so-called non-scalable size regime, where physical and chemical properties change non-linearly: melting point, ionization potential, geometric and electronic aspects can change with the addition or removal of a single atom. For some reactions, specific cluster sizes were found to be particularly selective or active catalysts: Pd_3_ clusters on TiO_2_ oxidize CO more readily than Pd_4_ or Pd_2_,^7^ while Pt_15_ clusters produced CO_2_ at twice the rate of Pt_14_.^8^ In another study, Pt_9_ was found to exhibit negligible activity for the ethylene hydrogenation to ethane, while Pt_10_ shows significant turnover under the exact same conditions^9^ – a single atom again makes a huge difference.

An aspect that is particularly difficult to grasp experimentally, yet significant for catalyst stability and activity, is their inherent dynamics during a reaction: structure, chemical composition, charge and oxidation state change continuously.^10^ On the one hand, clusters and nanoparticles deactivate by sintering, which needs to be mitigated with stabilization and redispersion strategies.^11–14^ On the other hand, the dynamic structural fluxionality of clusters has been invoked as the origin of the excellent catalytic activity of specific cluster sizes^15,16^ and might be tuned by the appropriate choice of the support. Dynamical modulation of support properties could then switch between competing catalytic pathways, a strategy proposed to overcome volcano-type limitations.^17^ Capturing dynamic transformations in real time and linking them to performance is thus essential for rational catalyst design.

In the fundamental studies on cluster surface chemistry presented here, we employ size-selected metal clusters as model catalysts. Inherently, their use allows identifying the most active cluster size with atomic precision.^18–20^ Using atomically resolved microscopy like scanning tunnelling microscopy (STM), classes of structural isomers of clusters can be distinguished.^11,21–23^ Moreover, the onset of sintering can be identified much more precisely than for samples with broad size distributions, since small changes become visible early on, thus providing detailed insights into sintering mechanisms, and by choosing a particular cluster size, one can even suppress certain sintering mechanisms.^24–26^ But particularly when using non-local, integral spectroscopy techniques, the *a priori* knowledge of cluster size is crucial to identify size-dependent phenomena.^20,22,27^ Size-selected clusters have been shown to exhibit a binding energy (BE) shifted from the bulk position in XPS due to final-state effects and do not yet exhibit metallic character, resulting in more symmetric peak shapes.^4–6^

Apart from the metal particles, the support plays a decisive role in catalysis. Non-reducible oxides are rather inert and rigid in their stoichiometry, but their Lewis acidity or basicity (*i.e.* capability to accept or donate electrons, respectively) and thus their work function can tune activity: in the case of ethylene hydrogenation on Pt mentioned above, activity trends were found to be reversed on an acidic SiO_2_*vs.* a basic MgO support, and also on thin film SiO_2_ surfaces differently doped by support work function tuning.^9,28–30^ Reducible supports can change their stoichiometry and participate more directly in reactions. Ceria, CeO_2_ – a common oxidation catalyst support – acts as an oxygen reservoir *via* formation of oxygen vacancies that are charge-balanced by reduction of nearby cerium ions.^31–33^ Metal particles on ceria have been shown to nucleate at O vacancies,^34,35^ and even the redispersion and formation of Pt clusters is feasible on ceria upon redox cycling.^14,36,37^ In contrast, magnetite, Fe_3_O_4_, is a support with high cation mobility, where Fe interstitials diffuse from and to the surface, and their subsurface mobility can be observed, as exemplified by Fe-rich defects.^38–41^ In the subsurface cation vacancy-reconstructed Fe_3_O_4_(001) surface, the surface stoichiometry is stabilized even under reducing and oxidizing conditions in UHV.^11,42^ Titania, TiO_2_, combines O vacancies and interstitials. Strong metal–support interaction (SMSI) effects^43,44^ have been first observed in this system, including charge transfer from support to particles,^45,46^ deactivation for molecular adsorption *via* encapsulation by a thin, sub-stoichiometric oxide layer,^47–52^ and the deep burial of particles in oxidizing conditions.^50^

A central challenge, and key focus of this paper, is how to control individual parameters of such complex systems.^10,50,53^ To follow catalyst evolution, we need precise starting points for our samples. While temperature and pressure can routinely be set precisely in experiments, it is non-trivial to change the cluster charge state, cluster dimensionality, support acidity and cluster–support interface individually. We therefore need sophisticated experiments to follow the evolution of clusters during sintering and redispersion as well as dimensionality change. In this context, synchrotron-based, high-resolution XPS is able to access such fundamental properties in a systematic way, irrespective of support roughness. Mass filters in laser ablation or sputtering cluster sources select clusters of a specific size with atomic precision^54–56^ to produce ‘naked’, ligand-free metal clusters for size-dependent characterization and reactivity studies, which we place onto flat, often single crystalline oxide supports where we precisely control defect densities and stoichiometry.^50,57^ Beyond the experimental precision, a further advantage of such highly defined models is that they can be rationalized in detailed theoretical modeling. Bringing such oxide-supported size-selected clusters to synchrotrons without the need for a dedicated cluster source on site^58^ vastly broadens the scope of accessible methodologies for robust and reproducible *in situ* and *operando* investigations.^22,59–66^ In particular when exchanging samples between different experimental stations, contaminants, defects in cluster supports, and gas environments must be known and controlled.

In the present paper, we report on three (NAP-)XPS investigations of Pt_*n*_ clusters (with *n* the number of atoms) at synchrotrons, where clusters were transported intact in air and in a vacuum suitcase, supplemented by our prior work with lab-based XPS and scanning tunnelling microscopy (STM). We discuss the evolution of Pt clusters on a range of different supports and in different environments. Our report is structured by different aspects of complexity: first, we control cluster charge by placing our model system Pt_*n*_/SiO_2_ on differently doped Si(100) wafers and show that the charge state of the support changes the clusters’ response to oxidizing and ammonia oxidation reaction conditions up to 0.7 mbar pressure, which we hypothesize as being due to a difference in cluster wetting of the support. Second, we discuss Pt_*n*_/CeO_2_(111)/Rh(111) where cluster dimensionality can be tuned *via* the concentration of O vacancies on CeO_2_, and clusters redisperse or sinter, depending on the temperature and gas environment. Finally, we show the evolution of Pt_*n*_/Fe_3_O_4_(001) – a support with rich structural dynamics – under 0.1 mbar H_2_ and O_2_ at room and elevated temperatures and discuss these changes on the background of SMSI-induced encapsulation and deep burial on TiO_2_(110).^50^

## Methods

Size-selected Pt_*n*_ clusters were prepared in a laser ablation cluster source (Pt target 99.95%, Goodfellow) using a 100 Hz Nd:YAG laser (Innolas Spitlight DPSS 250).^18^ The ablation plume was collision-cooled in a He pulse (8 bar, He 6.0) expanded through a nozzle to nucleate clusters, which were guided by ion optics into a quadrupole mass filter (Extrel 150-QC) for atom-precise mass selection. Soft-landing conditions were ensured by applying a retarding field, keeping the deposition energy below 1 eV per atom in the cluster. The cluster coverage on the sample was determined by integrating the neutralization current of deposited positively charged clusters (assumed singly charged). The cluster beam exhibited a Gaussian spatial distribution with a FWHM of ∼5 mm.

### Pt_*n*_/SiO_2_/Si experiments

Polished Si(100) wafers (CrysTec, Germany) with a thickness of 525 µm were used as supports, either phosphorus-doped n-type (0.015–0.025 Ω cm) or boron-doped p-type (0.01–0.05 Ω cm) Si, which we call n-Si and p-Si for short, respectively. These conductivities equate to dopant concentrations of ∼10^18^ cm^−3^. The wafers were cleaved into ∼10 mm × 10 mm pieces along the crystal orientation and cleaned in acetone and isopropanol. The native oxide was removed by etching in 5% hydrofluoric acid (HF) for 1 min, followed by rinsing in Millipore water. By exposure to air for 95 h, a thin SiO_2_ layer was regrown, with a controlled thickness of ∼1 nm. Pt_*n*_ clusters were deposited onto the SiO_2_/Si samples under high vacuum to a coverage of 2.0 atoms per nm^2^ and the samples subsequently moved through air into a glovebox, with only a few minutes in air. In the glovebox, they were packed into two nested click-boxes to minimize further oxide layer growth during transport.

XPS measurements were carried out at beamline 11.0.2 of the Advanced Light Source (ALS), Berkeley, USA, equipped with a SPECS Phoibos 150 electron energy analyzer. The samples were mounted on a boron nitride heater and the temperature measured with a type K thermocouple on the sample. Chamber base pressure was ≤5 × 10^−9^ mbar. The spot size on the sample was ∼200 µm and XPS spectra were acquired in normal emission, with 20 eV pass energy, 0.05 eV step size and 100 ms dwell time. To mitigate beam effects, spectra were acquired on fresh positions on the same sample by moving between every spectrum. The Pt 4f and Si 2p regions were measured with a photon energy *E*_phot_ = 278 eV and the C 1s region with *E*_phot_ = 490 eV, thus probing electrons with comparable kinetic energy. The same regions and survey scans (see SI, Fig. S1) were regularly measured using *E*_phot_ = 735 eV for each sample and new measurement condition. No significant contaminants other than C from transport were observed. The resolution under the chosen conditions was 0.1–0.3 eV. To clean the Pt_*n*_/Si samples, C contaminants were largely removed in 2.5 mbar O_2_ at 400–420 K for 20 min, while monitoring the Si 2p and/or C 1s regions. Ammonia oxidation experiments were conducted using low pressure O_2_ (typically 4 × 10^−5^ mbar, 5.0, Praxair) and NH_3_ (1 × 10^−5^ mbar, >99.98%, Sigma-Aldrich), while running temperature ramps up to 423 K, and reference checks in NO (>98.5%, Sigma-Aldrich) and in 2% O_3_ in O_2_ using an ozone generator. We further note that we have observed C deposition by the synchrotron beam.

Peak fitting was performed in KolXPD (v1.8.0)^67^ using Shirley or linear backgrounds. Spectra were normalized to the low BE background, and all binding energies were referenced to the Si^0^ component at 99.3 eV, measured with *E*_phot_ = 278 eV (since adventitious carbon was not a stable reference upon redox cycling at elevated temperatures). To this purpose, Si 2p core level peaks were fitted according to Himpsel *et al.*,^68^ with an additional β-phase. Pt 4f spectra were fitted with Doniach–Šunjić line shapes convoluted with a Gaussian. The spin–orbit splitting was fixed to 3.32 eV and the Lorentzian width to 0.25 eV, while the Gaussian width (and, where applicable, the asymmetry) was allowed to vary to account for cluster restructuring effects; the Pt 4f_7/2_ : Pt 4f_5/2_ intensity ratio of 1.4–1.5 deviated slightly from the nominal value of 4/3 due to background artifacts.

For checks of Pt_*n*_ cluster size *via* scanning transmission electron microscopy (STEM), clusters were deposited onto TEM grids with 8 nm thin suspended Si_3_N_4_ membranes (TedPella, layout see SI, Fig. S2), using a hot filament in the vicinity for charge compensation on the sample. These samples were transported in air. STEM was performed on a probe-corrected FEI Titan Themis in high-angle annular dark-field mode (STEM-HAADF) at 300 kV.

### Pt_*n*_/CeO_2_ experiments

For the methods used in the Pt_*n*_/CeO_2_(111)/Rh(111) experiments, we refer the interested reader to the original publication.^57^ The binding energy scale of the lab-based XPS was calibrated by setting the Rh 3d_5/2_ peak to 307.2 eV.

### Pt_*n*_/Fe_3_O_4_ experiments

Natural Fe_3_O_4_(001) crystals (SurfaceNet GmbH) were mounted on flag-style sample holders and prepared by Ar^+^ ion sputtering (20 min, 4 × 10^−5^ mbar Ar, 1 keV) and annealing in an oxygen atmosphere (20 min, 5 × 10^−7^ mbar O_2_, 983 K). Pt_10_ clusters were soft-landed onto the samples in UHV at room temperature (RT) up to a coverage of 0.5 atoms per nm^2^. Since magnetite is more susceptible to changes during interaction with air than silica, the samples were transported to the MAX IV Laboratory in Lund, Sweden, in a home-built UHV vacuum suitcase with a constant pressure of ∼1 × 10^−10^ mbar.

NAP-XPS measurements were performed at the HIPPIE beamline at MAX IV, equipped with a Scienta Omicron HiPP-3 electron energy analyzer, using a photon energy of *E*_phot_ = 921 eV. The samples were heated with an infrared laser directly illuminating the back, and the temperature was measured with a type K thermocouple mounted to the side of the crystal. We indicate the temperatures as measured, which could be somewhat underestimated. XPS spectra were acquired in normal emission and with constant pass energy. Initial checks showed no significant beam damage; thus we recorded all spectra at the same spot on the sample (*i.e.* with the same cluster coverage) to allow quantitative comparison of the spectra. To desorb any adsorbates accumulated during the several-day-long transport in the UHV suitcase, we initially heated each sample to 373 K, resulting in a shift of 0.6 eV to lower binding energies (likely due to CO desorption) and a sharpening of the Pt 4f signal. O_2_ and H_2_ were introduced into the reactor cell using mass flow controllers.

Peak fitting was performed in KolXPD using a Shirley background. Spectra were normalized to their low BE background. The binding energies were calibrated to the Fe^3+^ 3p signal (see SI, Fig. S3) from deconvolution, resulting in an uncertainty of ±0.1 eV, judging by the Fermi edge (see SI, Fig. S4) of samples in the reduced state.

## Results and discussion

We present the evolution of size-selected Pt_*n*_ clusters on three distinct supports, SiO_2_, CeO_2_(111) and Fe_3_O_4_(001), in the three sections below. In each, we focus on a different aspect of complexity: on silica, we discuss how the charge state of the support tunes cluster dimensionality and reactivity. On ceria, we explore cluster dimensionality control by the reduction state of the support and consider stabilization and self-repair strategies. Finally, on magnetite, we study how the rich support dynamics of in-depth UHV studies^11,23,26,38,40,69,70^ translate into higher pressure regimes and compare them to titania, the prototypical system for SMSI-induced effects.^43,44,48–50,52^

### Evolution of size-selected Pt_*n*_ clusters on a non-reducible support: Lewis acidity and cluster charge state

**Fig. 1:**
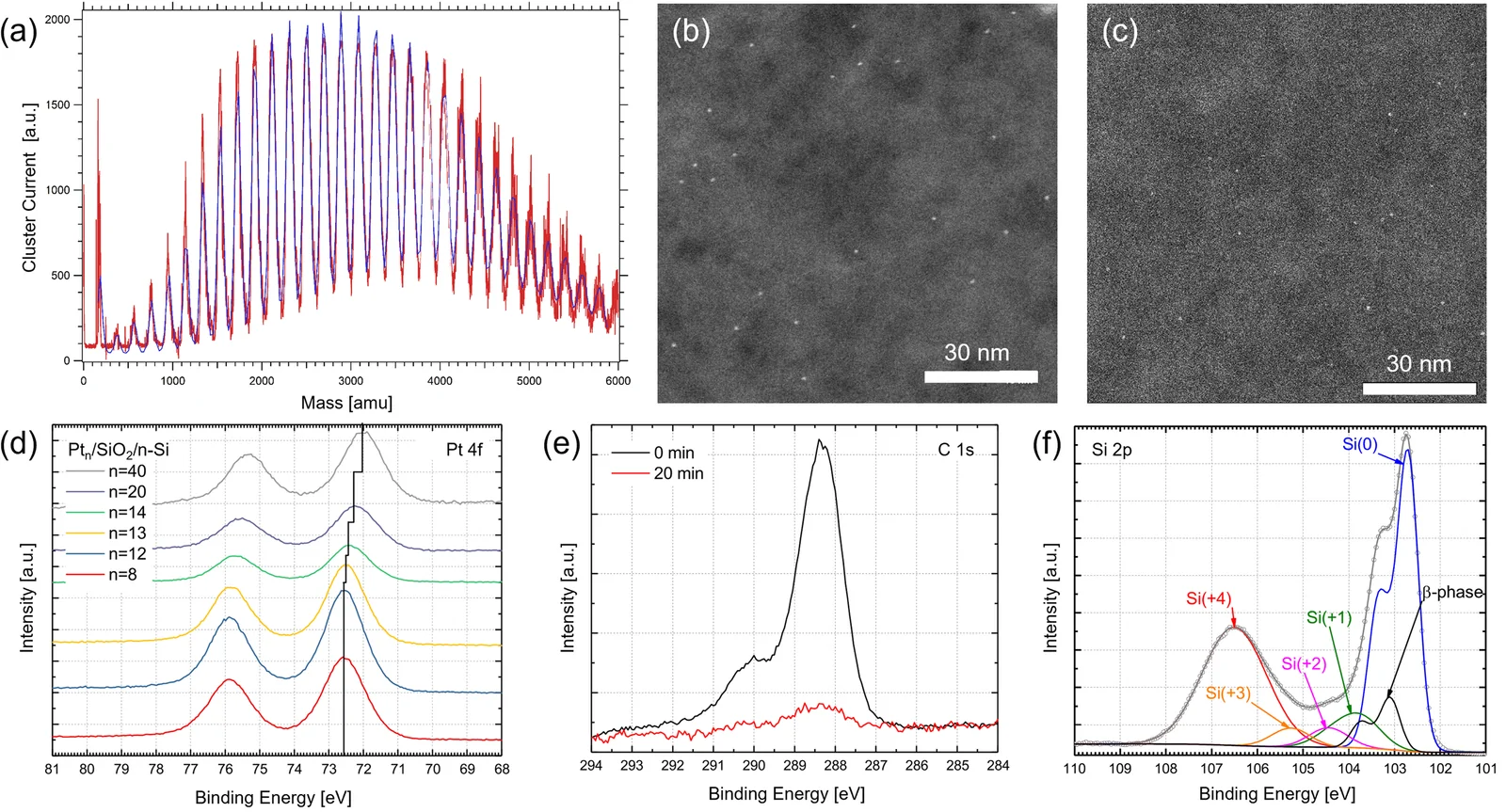
Size-selected Pt_*n*_ cluster generation, deposition and transport on silicon nitride and silicon oxide supports. (a) Scanning the mass-to-charge ratio *via* the quadrupole mass filter gives a mass spectrum with an intense peak for Pt atoms and separate peaks for Pt_*n*_ (*n* = 2, 3, 4, … 31) clusters. (b and c) Pt_20_ clusters deposited onto the 8 nm thin Si_3_N_4_ membrane of a TEM grid imaged *via* HAADF-STEM (b) before and (c) after transport from Munich to Berkeley in air, XPS measurements in oxidizing and reducing atmospheres and transport back to Munich. (d) Pt 4f region of Pt_*n*_/SiO_2_/n-Si(100) samples with *n* = 8, 12, 13, 14, 20 and 40 atom clusters. The black vertical line is a guide to the eye, illustrating the binding energy size dependence. (e) C 1s region of a SiO_2_/n-Si sample taken at 490 eV during sample cleaning in 0.7 mbar O_2_ at 393 K, at the beginning of the treatment (black) and after 20 min (red). (f) Representative Si 2p spectrum and fit.

Fig. 1 lays out the experimental sequence that underpins all subsequent experimental interpretation: the clusters must be deposited size-selected, remain intact during transport, and be brought back to a clean, well-defined state inside the NAP-XPS chamber before reactivity trends are investigated. The mass spectrum of Pt_*n*_ clusters generated by the size-selective cluster source in Fig. 1a shows an intense peak for Pt atoms and a peak for each Pt_*n*_ cluster from *n* = 2 to *n* = 31 atoms. The peaks are separated such that true size selection is achieved by setting the quadrupole mass filter to the peak corresponding to the desired cluster size. To test the feasibility of atom-precise deposition of Pt_*n*_ clusters onto silica supports and subsequent transport, we employed HAADF-STEM imaging on clusters deposited onto TEM grid-supported, 8 nm thin Si_3_N_4_ films. These films are sufficiently thin for high resolution imaging of small sub-nm clusters. Our XPS measurements showed that the nitride films are oxidized upon air exposure at RT, which a past study interpreted as the formation of a nanometer-thick, oxygen-rich surface oxynitride.^71^ Their oxidic surface makes the interface between Pt_*n*_ clusters and TEM grids chemically sufficiently similar to that on the SiO_2_/n-Si samples to be a suitable test case for cluster stability checks. We transferred the TEM grid-supported Pt_*n*_ clusters through air into a STEM, transported them to the ALS for NAP-XPS experiments with O_2_ and H_2_ partial pressures of up to a few millibar, and subsequently back to Munich for HAADF-STEM imaging. Fig. 1b and c show representative images of the same Pt_20_/Si_3_N_4_ sample before and after the beamtime, respectively. In both cases, a sparse distribution of equally sized small white specks is visible – the Pt_20_ clusters. We found no evidence that the clusters changed size during any of the treatments. Due to the high local electron dose required to image these tiny particles on an 8 nm nitride film, the clusters seemed to disintegrate in higher-resolution HAADF-STEM images (not shown); thanks to the sparse distribution, this actually allowed us to count the number of atoms and confirm the size selection. Note that the cluster coverage in the experiments presented in the following is around 4 times higher than that shown in the STEM images, but still low enough to ensure cluster separation and a statistically insignificant number of cluster multimers. The low coverage on the TEM grids is a result of the poor conductivity of the ultrathin nitride films, leading to repulsion of the positively charged clusters upon deposition. Indeed, raster-scanning the TEM grids laterally while recording the Pt 4f signal confirms a lower signal intensity in the location of the nine holes covered only by the suspended membrane compared to the regions supported by silicon (see SI, Fig. S2).

For the next beamtime on Pt_*n*_/SiO_2_/Si, we thus deposited a coverage of 2.0 atoms per nm^2^ of Pt_*n*_ clusters onto an approximately 1 nm thick native oxide film on strongly doped, highly conductive Si(100) wafers. The overwhelmingly large O signal from the SiO_2_ support precludes useful analysis of the O 1s region to understand O coordination to the clusters and we thus only discuss Pt 4f peaks in the following. Several representative Pt 4f spectra of clusters between 8 and 40 atoms deposited on n-type Si samples are shown in Fig. 1d, illustrating two essential characteristics of sub-nm clusters. Note that these spectra are referenced to the Si^0^ component. Band bending effects in silicon do influence the reference, but depth profiling indicates it to be <0.1 eV. The small effect of band bending is expected for spaced metal clusters on heavily doped semiconductors that cannot uniformly pin the Fermi level [cite https://doi.org/10.1103/PhysRevB.43.11806] and further reinforced in our system by the insulating 1 nm oxide layer that charges need to tunnel through and the sub-nm cluster size with a low density of states near the Fermi level. This leaves the bulk Si^0^ 2p peak as a conceptually imperfect reference, but we find it sufficiently reliable and consistent across data from four beamtimes and many different samples. First, it is clearly visible that the Pt 4f peak of such small, sub-nm species is much more symmetric than that of bulk-like metallic Pt. For all cluster sizes investigated here, the spectra of the initial samples could be fitted satisfactorily by a single Doniach–Šunjić doublet of small asymmetry (∼0.05) convoluted with a Gaussian. Second, the Pt 4f BE shifts by a total of 0.6 eV to higher binding energies with increasing cluster size (discussed in more detail below), which is an electrostatic final state effect, in good agreement with prior reports.^4–6^

Before investigating the effects of gas atmospheres on these clusters, we need to ensure their surface is actually accessible to reactants. Unsurprisingly, the samples show a significant adventitious carbon peak after transport in air and introduction into the vacuum chamber at the beamline. Therefore, we have developed a cleaning procedure where the carbon signal was largely removed in an oxidative treatment in 0.7 mbar O_2_ at ∼400 K. Fig. 1e shows the C 1s region of an SiO_2_/n-Si sample at the beginning (black) and after 20 min of the cleaning treatment (red), where the largest part of the C signal is removed. In subsequent tests, we found that any residual oxygen is removed from the Pt clusters after the oxidative treatment by evacuation to 10^−9^ mbar pressures, and additional reduction by heating in CO does not further shift the Pt 4f. The clusters after the cleaning process and pumping out of O_2_ are thus in the same chemical state as after deposition. Inelastic mean free path (IMFP) analysis of Si 2p spectra, using an IMFP of 0.963 nm for Si and 1.198 nm for SiO_2_,^68,72^ indicates an irreversible thickness increase by ∼0.4 nm of the silica layer during the oxidative treatment (see SI, Fig. S5), yet systematic checks of the Pt 4f region showed no significant change of the clusters. This simple protocol thus constitutes a reproducible way to return the surface to a clean state without significant changes to the chemical state of the sample nor cluster sintering (since the binding energy distribution of the Pt cluster samples remains).

Finally, Fig. 1f illustrates peak fitting of the Si 2p region. A sharp Si^0^ doublet is shown in blue, which results from the bulk Si wafer underneath the thin oxide film and clusters. Furthermore, four peaks for the four oxidation states of SiO_*x*_ and an additional β-phase are fitted using a standard routine from the literature.^68,73,74^ The fact that three peaks for intermediate oxidation states must be included in the fit shows that the native oxide is not a perfect SiO_2_ layer, but contains sub-oxide species – likely at the interface between Si wafer and oxide film. In this work, we use the fit solely for energy referencing to the Si^0^ peak, which is robust against minor changes in the oxide components.

Taken together, the experiments shown in Fig. 1 establish that the size-selected clusters can be transported through air while maintaining their number of atoms, that the contamination from transport can be removed by an oxidative cleaning step and that we have a robust binding energy referencing routine.

**Fig. 2:**
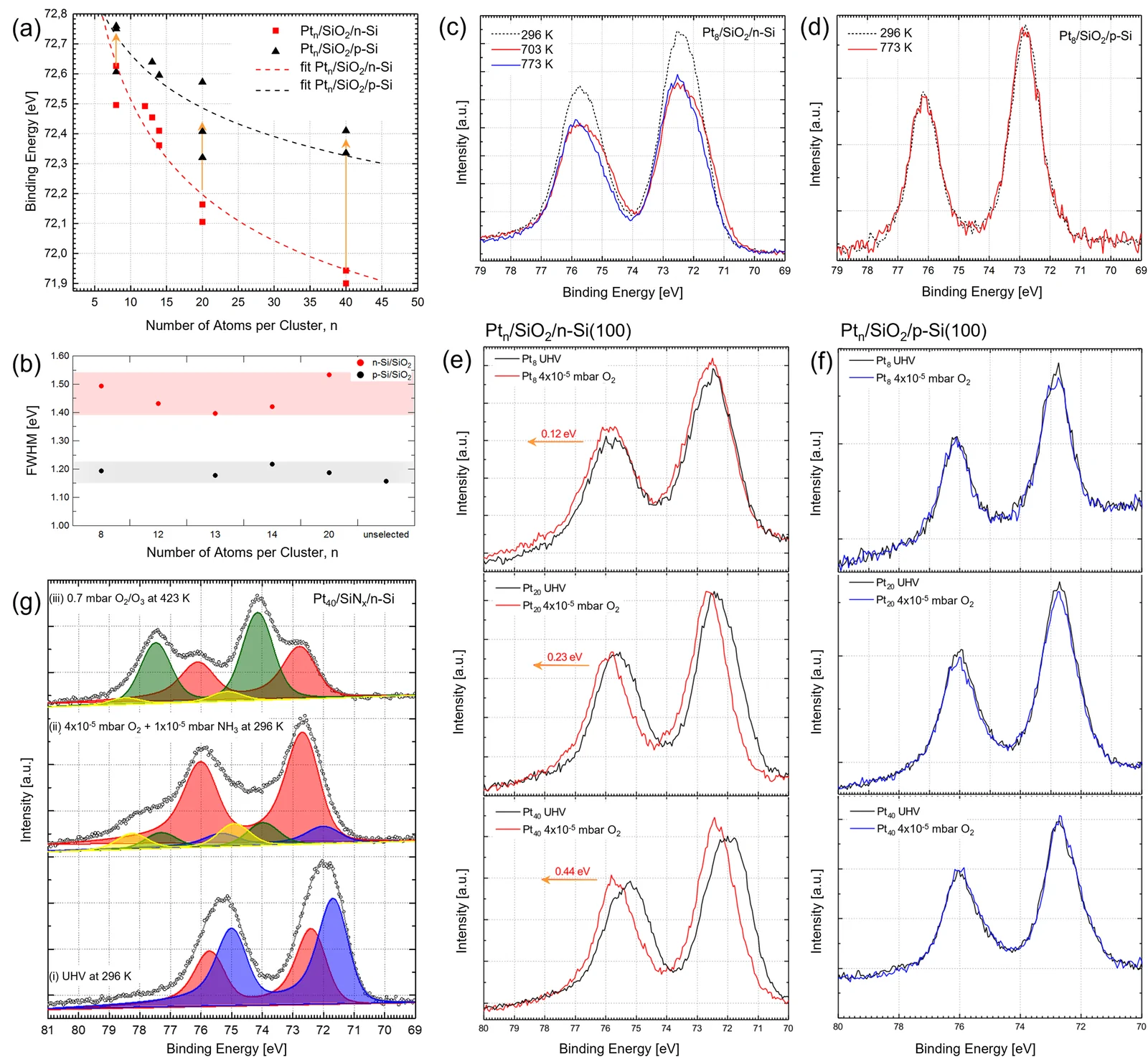
Evolution of Pt_*n*_ clusters on SiO_2_ on differently doped Si(100) in oxidizing and reaction environments. (a and b) Cluster size-dependence of (a) the binding energy and (b) the FWHM of the Pt 4f_7/2_ peak for Pt_*n*_/SiO_2_/n-Si (red) and Pt_*n*_/SiO_2_/p-Si (black) as deposited, with red and black dashed lines showing respective fits of the Wertheim model. The orange arrows correspond to the shifts shown in (e). (c and d) Sintering study of Pt_8_ on (c) SiO_2_/n-Si and (d) SiO_2_/p-Si up to 773 K. (e and f) Pt_8_, Pt_20_ and Pt_40_ on (e) SiO_2_/n-Si and (f) SiO_2_/p-Si in UHV (black) and during exposure to 4 × 10^−5^ mbar O_2_ (red/blue). (g) Pt_40_/SiO_2_/n-Si in (i) UHV at RT, (ii) a mixture of 4 × 10^−5^ mbar O_2_ and 1 × 10^−5^ mbar NH_3_ at RT, and (iii) a mixture of O_2_ and O_3_ with a total pressure of 0.7 mbar at 423 K. Peak fitting with four components is shown as colored peaks.

We now have the practical foundation to investigate the evolution of the Pt clusters on two differently doped wafers and in different gas atmospheres, shown in Fig. 2. As mentioned in the introduction, previous work by some of the authors showed that acidic and basic supports can alter the activity of Pt_*n*_ clusters of identical size by withdrawing respectively donating electrons from/to the cluster.^9,30^ In order to clearly disentangle the effect of support chemistry and cluster charge state, we have employed a somewhat different strategy in this work: we have deposited the same clusters onto the native oxide of phosphorus-doped n-type (‘n-Si’) and boron-doped p-type Si wafers (‘p-Si’), of similarly high conductivity, to tune the Lewis acidity *via* bulk properties. The Si 2p peak shapes and the oxide layer thicknesses are similar for samples of both types (see SI, Fig. S5).

Fig. 2a shows the binding energy of the Pt 4f_7/2_ peak for Pt_*n*_ clusters from 8 to 40 atoms in size on SiO_2_/n-Si (red) and SiO_2_/p-Si (black), obtained by fitting a single component. Strikingly, the binding energy depends significantly more strongly on cluster size for n-type than for p-type samples. Fitting the two data sets with an empirical function proportional to *n*^−1/*c*^ (with *n* the number of atoms and *c* an empirical constant) on SiO_2_/n-Si (red line) and SiO_2_/p-Si (black line) returns 1/*c*_n-Si_ = 1/2.9 = 0.34 and 1/*c*_p-Si_ = 1/6.1 = 0.16, respectively. The result for p-Si agrees well with a previous report for Au clusters on an SiO_2_/p-Si support described by Peters *et al.*,^6^ where a value of 0.12 was measured experimentally. The authors compare this result to an expected value of 0.33 for spherical free clusters, based on a final state charge model that assumes metallic behavior and takes into account quantum corrections, and an exponent of 0.5 for a simple electrostatic drop model.^75,76^ They further assign the weaker size dependence of the BE to faster support-induced core–hole screening, thus a more intimate charge transfer between clusters and the support. In contrast, the binding energies of the Pt_*n*_/SiO_2_/n-Si samples align with the picture of free (hemi)spherical 3-dimensional (3D) clusters, pointing to a weak interaction. Our findings indicate that the charge transfer between cluster and support depends on the type of doping.

Perhaps surprisingly, the BEs relative to Si^0^ converge in the small-scale limit but there is an offset when extrapolating to the bulk, where size-dependent final state effects disappear. To assign the origin of this offset, we consider three further observations: (i) the full width at half maximum (FWHM) of Pt clusters on SiO_2_/p-Si is narrow compared to clusters on SiO_2_/n-Si (see Fig. 2b), which indicates that all atoms on p-type samples have a similar environment. (ii) All Pt clusters are oxidized: the total BE shift relative to bulk Pt^0^ 4f 7/2 (71.2 eV) exceeds typical maximum size-induced shifts of up to 0.8 eV. (iii) The binding energy offset vanishes when n-type samples are exposed to low pressure O_2_ (see orange arrows in Fig. 2a and data for the three representative cluster sizes Pt_8_, Pt_20_, Pt_40_ in Fig. 2e and f), which indicates a reversible reduction/oxidation process occurring on n-type samples. While the exact oxidation state of sub-nm clusters is difficult to pinpoint,^58^ we find that the magnitude of the offset increases with cluster size (≈0.12 eV for Pt_8_, ≈0.23 eV for Pt_20_, and ≈0.44 eV for Pt_40_). In all cases, the shift is small enough to suggest only O adsorption or at most partial oxidation. We tentatively rationalize these observations with the picture of 2-dimensional (2D) clusters, or wetted islands, on p-type, and 3D clusters on n-type samples. Simply speaking, the addition of more Pt to a 2D island (*i.e.* going to larger cluster sizes) extends the lateral size of the islands, while keeping the interaction with the support the same for all atoms. The n-type supported clusters, in contrast, are more similar to free clusters, have a stronger footprint dependence, and contain atoms in different environments (hence the larger FWHM and the need to fit with two doublets); this difference is lifted when oxygen binds to the surface atoms of the (hemi)spherical clusters.

Since charge transfer from the clusters to the support must be responsible for the offset, it will be smaller for smaller cluster sizes for which ionization potentials increase.^77^ This explains why the data converges for smaller clusters. The doping increasingly matters for larger clusters, where charging costs less energy. In our specific case, the electron-accepting support (SiO_2_/p-Si) can then take up charges from Pt, while the electron-rich support (SiO_2_/n-Si) cannot. However, if O_2_ is adsorbed on the particles, it facilitates the electron uptake and Pt 4f of the clusters shifts to the same positions as for the clusters on SiO_2_/p-Si (indicated by arrows in Fig. 2a and e). The specific charging on the p-type support could explain their 2D morphology, similar to image charge-induced flattening of Au clusters on thin films of MgO on Ag(001).^78^ As we will discuss in the next section, we have recently reported a different type of 3D *vs.* 2D cluster dimensionality change for Pt_*n*_/CeO_2_(111) caused by mobile surface defects – another route to accommodate interfacial Pt.

Conversely, the Pt 4f line shape can be used to monitor changes in the cluster distribution during sintering. Fig. 2c and d show the Pt 4f spectra for Pt_8_ clusters on the two supports heated to 773 K in UHV, respectively. In the case of the n-type support, a clear signature of sintering is visible around 700 K. The overall peak intensity decreases, indicating a lower surface-to-bulk ratio, and the peak shape broadens, evidence of a wider distribution of Pt species. In contrast, the Pt 4f peak of the same Pt_8_ clusters on SiO_2_/p-Si does not change up to 773 K, indicating that the strongly wetting Pt_*n*_ clusters on SiO_2_/p-Si are exceptionally sinter-resistant.

In practical terms, this means that the same cluster size does not necessarily lead to the same cluster dimensionality and chemistry, which instead depend on the doping of the support, even when the cluster–support interface is chemically similar. Therefore, we next look into how differences in cluster charge state and dimensionality affect their reactivity. We chose the ammonia oxidation reaction as a technologically relevant and mechanistically rich model reaction, which is known to result in significant structural changes of the catalyst and occurs already under mild conditions on stepped Pt surfaces.^79,80^ While we have recorded the N 1s spectrum for all conditions, reference measurements on a bare SiO_2_/Si support showed that small amounts of N-containing species also adsorb on silica, thus making it impossible to attribute species to the clusters; we thus again restrict ourselves to studying the Pt 4f region. Fig. 2g (bottom spectrum) shows the example of Pt_40_ clusters on SiO_2_/n-Si. After initial oxidative annealing, pumping back to UHV and cooling to RT, the Pt 4f region could be fitted with two components (blue and red peaks). Note that the clusters in the as-introduced samples mentioned above are adsorbate-covered, which might explain why all peaks could be fitted with a single doublet (most likely corresponding to the red peak). Upon exposure to a reaction mixture of 4 × 10^−5^ mbar O_2_ and 1 × 10^−5^ mbar NH_3_ at RT, four distinct components can be distinguished, marked by four differently colored doublets in Fig. 2g. These spectra could not be mimicked by exposure to NO – a typical reaction intermediate – nor could they be reproduced by O_2_ or NH_3_ separately (see SI, Fig. S6 and S7), suggesting that the new peak components are not due to simple adsorption of educts or common intermediates on Pt. In contrast to the experiments described above, significant beam effects were observed in the reaction mixture. Specifically, the Pt 4f components at higher binding energies were depleted in the beam. We could circumvent this effect by introducing a waiting time with the beam shutter closed for 6 min to replenish the oxidized species before recording each spectrum as quickly as possible.

Since the binding energies for clusters of different oxidation states are not tabulated and in general difficult to assign,^58^ we have exposed the same sample to a strongly oxidizing environment by dosing 2% ozone in 0.7 mbar O_2_ at 423 K. In these harsh conditions, the main peak shifted by approximately 1.5 eV to higher binding energies, indicating a change due to real Pt_*n*_ cluster oxidation (top spectrum, Fig. 2g). Fitting all three spectra (in UHV, reaction mixture and O_3_/O_2_) allows us to assign the four components: the blue component around 71.8 eV is the main component after cleaning and its intensity decreases in the reaction environment; we thus assign it as the surface core level shift of clean Pt atoms. In the same line, we interpret the red component around 72.5 eV as Pt atoms in contact with the SiO_2_ support or bound to chemisorbed O species. Looking back at the Pt_40_ sample exposed to low pressure O_2_ in Fig. 2e, this fits with our previous interpretation of O adsorbates on the clusters. Clean 3D Pt_40_ clusters thus contain two components, from the interface atoms and the ones at the surface. The two higher binding energy components (green and yellow) at about 74.1 eV and about 75.0 eV suggest a 2.3 eV and 3.2 eV shift, respectively, with respect to the clean cluster state and must therefore be assigned to two higher oxidation states (most plausibly Pt^2+^ and Pt^4+^), consistent with their appearance during ozone treatment. We can thus conclude that ammonia oxidation reaction conditions are oxidizing the clusters more strongly than a pure O_2_ environment, even in the strongly reducing conditions of the beam, making careful experimentation critical. One might speculate that the highly exothermic reaction can introduce sufficient energy into the clusters to induce a dynamic restructuring (fluxionality), making them more susceptible to oxidation. By contrast, the Pt 4f spectra of Pt_20_/SiO_2_/p-Si only exhibit a slight shoulder from oxidized species under the same conditions (see SI, Fig. S8), indicating reduced duster reactivity for these samples also under ammonia oxidation conditions.

Overall, this study demonstrates that size-selected cluster samples can be transported to synchrotrons intact and represent powerful samples for studying cluster restructuring, incipient sintering and cluster oxidation: the onset of particle growth or of support-induced electronic changes becomes visible as subtle, but distinct modifications in peak position, width and line shape. Once contamination from transport is removed, the Pt 4f region can be used as a quantitative signature across a sequence of increasingly realistic environments, setting the basis for later *operando* interpretation in true catalytic mixtures: the meaningful experimental observable is not a single binding-energy number, but the evolution of a distribution of Pt states that reflects the competition between cluster oxidation, charge transfer through the support, and thermally activated restructuring. The doping of Si wafers appears to change the Lewis acidity of the native silicon oxide film to create differently charged clusters of the same size and provides a platform for future fundamental *in situ* investigations on the influence of cluster charge on catalytic activity and for experimental investigations within catalytic resonance theory.^17^

### Evolution of size-selected Pt_*n*_ clusters on an O-vacancy-dominated reducible support: dimensionality, redispersion and sintering

We have recently reported a similar tuning of cluster dimensionality in a more complex system, namely Pt_*n*_ clusters on a reducible CeO_2_(111) support that can actively participate in a reaction.^57^ In this system, O vacancies dominate the surface chemistry and cluster charge state – local effects thus come into play. In a quantitative STM and XPS investigation, we followed the dynamic cluster restructuring during sintering in UHV. The key findings are briefly described in this section and discussed in the context of Pt_*n*_ cluster evolution on a support that has attracted attention for its capability to reversibly redisperse and form catalytically active clusters under redox cycling, thus bearing great promise as a self-repairing system.

**Fig. 3:**
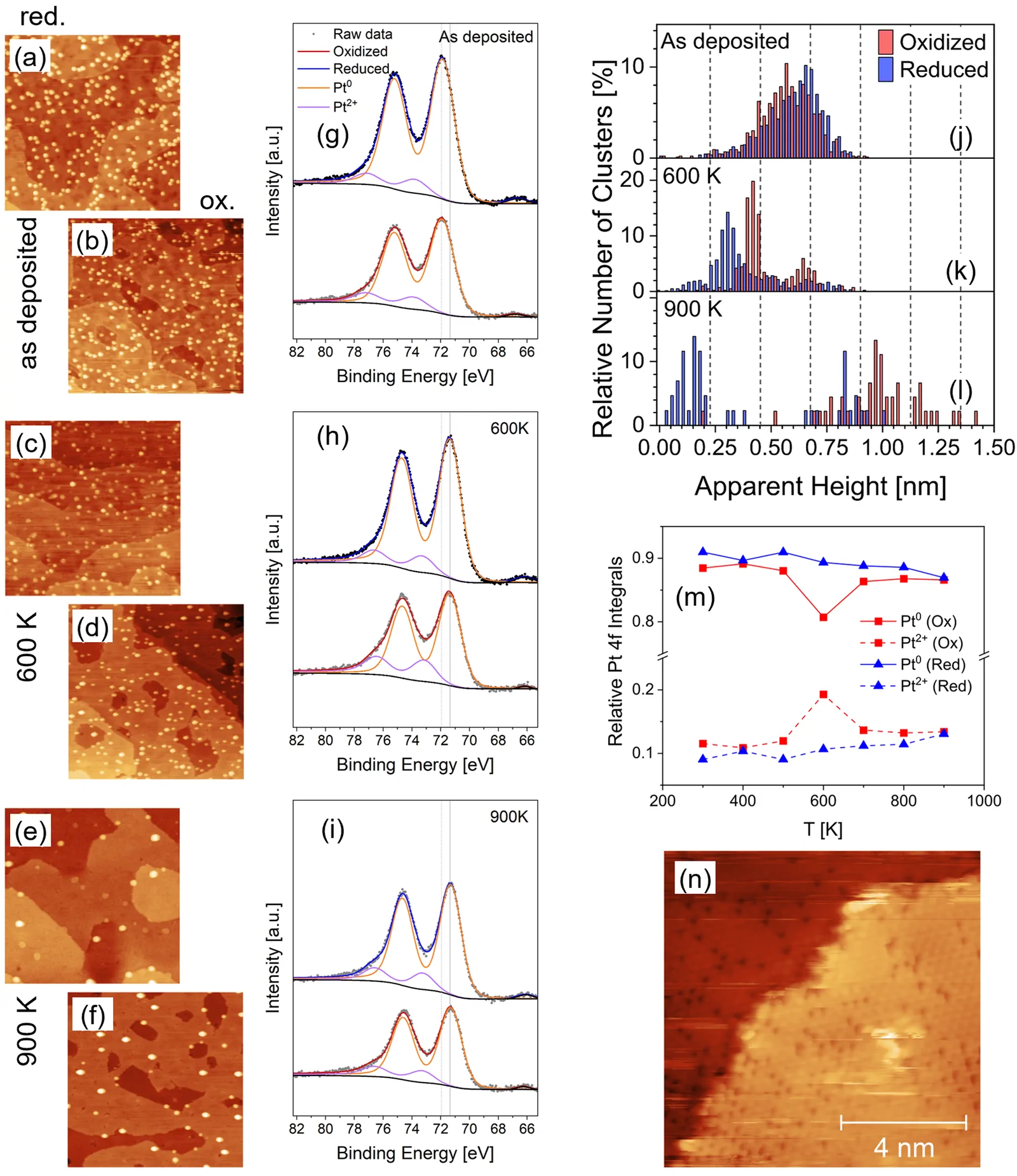
Evolution of Pt_20_ clusters on CeO_2_(111) during sintering in UHV. (a–f) 100 × 100 nm^2^ STM images of Pt_20_ clusters on (left) slightly reduced and (right) fully oxidized ceria at RT (“as deposited”) and after annealing to 600 K and 900 K. (g–i) Corresponding XPS spectra of the Pt 4f region with a tentative fit of Pt^0^ and Pt^2+^ species. Note that the binding energy scale was adjusted by 0.25 eV compared to the original publication. Start and end peak positions are marked with dashed and solid grey lines, respectively. (j–l) Histograms of the apparent cluster height from STM images for the same clusters on oxidized (red) and reduced (blue) ceria. (m) Relative Pt 4f peak integrals of the Pt^0^ and Pt^2+^ components from the XPS spectra in (g–i). (n) STM image of a slightly reduced ceria shows several O vacancies as dark spots. Reproduced under CC-BY license from ref. 57.

By evaporating cerium in an O_2_ atmosphere, single crystalline thin ceria films of controlled O vacancy number and thus stoichiometry were grown on Rh(111). Targeted redox cycling by annealing alternately in O_2_ and UHV resulted in defect-poor films with extended terraces, while gentle annealing in methanol reduced exclusively the top trilayer, without changing deeper ceria layers.^81^ The surface reduction state was quantified *via* grazing emission XPS measurements. Pt_20_ clusters were then soft-landed on two ceria thin films of distinct surface reduction state, one with CeO_2_ stoichiometry and one with a higher O vacancy density, translating to a CeO_1.94_ surface stoichiometry. Fig. 3 summarizes the temperature evolution of the clusters, with a coverage of 0.5 atoms per nm^2^. In the left column (Fig. 3a–f), representative STM images of Pt_20_ on the reduced (left) and oxidized (right) support at select temperature steps are shown: as deposited at RT, after annealing to 600 K and after annealing to 900 K. Height profile analysis of 10 large-scale STM images at each temperature gives histograms of the cluster size distribution under each condition (see. Fig. 3j–l). Notably, the histograms at RT perfectly match for the same clusters deposited onto ceria with different surface O vacancy density. This observation serves both as confirmation of the robustness of the method and provides the important fundamental insight that Pt_*n*_ clusters essentially adsorb as they land. Since the O vacancies are not yet mobile and statistically distributed, the clusters see essentially the same single vacancy environment on oxidized and reduced surfaces.

The corresponding XPS spectra of the Pt 4f region are presented in Fig. 3g–i. The Pt 4f BE of the Pt^0^ species shifts from around 71.7 eV to 71.2 eV upon annealing to 600 K and slightly further to 71.1 eV after annealing to 900 K on both the reduced and oxidized surface, comparable to findings reported by Lykhach *et al.*,^82^ ranging from clusters to larger nanoparticles on ceria films.^83–86^ The Pt 4f region can be fitted with a simple model containing two components, representing metallic Pt^0^ and cationic Pt^2+^ species (see Fig. 3m, obtained from fits with two components separated by 1.9 eV). While the exact area ratio strongly depends on fit constraints, the outlier at 600 K on the oxidized support is robust: at this intermediate temperature, steps on the oxidized surface take up Pt atoms but release them again once excess oxygen leaves the surface at higher temperatures.^83,86^

This support-independent cluster appearance quickly changes upon UHV annealing. We found the clusters to not only sinter *via* Ostwald ripening from about 600 K onwards, but comparing the STM images and height histograms on supports with different O vacancy numbers further indicates that their dimensionality changes drastically: while the height distributions spread out on both supports, only on the reduced CeO_1.94_(111) do the majority of particles appear to wet the support and assume a 2D island-like geometry (reflected as a peak between 0.1 and 0.2 nm in height in the histogram). Interfacial Pt atoms should thus be more abundant on reduced ceria. The XPS spectra, however, do not reflect this tendency. On both supports, the Pt 4f_7/2_ BE is 71.1 eV after annealing, which is expected for largely metallic Pt; also the line shapes are comparable and show similar asymmetry. The clusters thus retain their charges and the dimensionality change has to have another explanation. In the STM images we observe a high mobility and ordering of the O vacancies at the annealing temperatures – note the periodically arranged O vacancies appearing in Fig. 3n as dark depressions. This suggests that O vacancies accumulate at the interface to the flat clusters (Fig. 3l); their very low apparent height might even point to a partial cluster embedding. In this way, interfacial Pt gets stabilized and wets the surface as long as there are enough O vacancies available, *i.e.* on the reduced surface. This behavior is in stark contrast to the case of Pt_*n*_/SiO_2_/Si, where the BE deviates significantly for the same cluster size on the two supports. The fundamental difference here likely lies in ceria being reducible and thus competing with Pt for electrons, while SiO_2_ is inert in this regard. On ceria, local changes in the electronic structure below the clusters can thus possibly compensate for the shifts observed on SiO_2_.

This wetting behavior cannot be explained by a dispersion of the clusters into Pt atoms and their reformation into a new geometry, because nucleation-based growth cannot explain the identical cluster coverage at each sintering step on the oxidized and reduced ceria supports. A wetting of the originally deposited clusters must therefore occur *via* a change in cluster dimensionality during sintering. However, at the 600 K intermediate step, we do see evidence for temporary stabilization of atomic Pt species – capturing a transient state in the Ostwald ripening process and a temporary partial redispersion of Pt_*n*_ clusters. This is reflected in the increase of the Pt^2+^ signal in Fig. 3m.

Overall, the Pt_*n*_/CeO_2_ system presents another highly defined platform to explore the evolution of Pt_*n*_ clusters. Having laid the groundwork in this detailed UHV investigation, we anticipate this model catalyst to provide a versatile playground for investigating the catalytic activity, sinter resistance and structural dynamics of 3D *vs.* 2D particles. Especially in combination with the Pt_*n*_/SiO_2_ system discussed in the previous section, the same cluster size can now be compared on an inert, non-reducible and on an O vacancy dynamics-dominated reducible support in future *in situ* studies. A key question concerns whether cluster dimensionality can be switched dynamically and reversibly to tune the cluster catalytic activity and how the reversible, redox condition-triggered redispersion and cluster formation can be sustainably controlled to implement a true self-repair capability into applications of this supported catalyst.

### Evolution of size-selected Pt_*n*_ clusters on an interstitial-dominated reducible support: dynamics, encapsulation and burial

In this final section, we now turn to another type of reducible oxide support, namely one dominated by cation interstitials and their exchange with the bulk, rather than surface O vacancies. The magnetite, Fe_3_O_4_(001), surface has been described in detail in UHV, in terms of its intrinsic structural dynamics and its capability to stabilize single-atom catalysts.^12^ In our own previous work, we have disentangled the rich dynamics of Fe-rich defects *via* FastSTM at elevated temperatures^42,69^ and described the atomic-scale structural evolution of Pt_*n*_ clusters upon heating in UHV: lattice oxygen reverse spillover onto Pt_*n*_ clusters provides a highly active O species, resulting in etching of the surrounding support as Fe cations diffuse into the bulk to maintain the surface stoichiometry.^23^ However, this initial high activity in oxidation catalysis is quickly lost upon SMSI-induced encapsulation of the clusters. Remarkably, we have shown that encapsulated Pt_5_ and Pt_10_ clusters can still diffuse on the surface and coalesce to larger clusters, but atoms do not detach from these encapsulated clusters, suppressing Ostwald ripening up to a surprisingly high temperature of 823 K – a sintering onset that is ∼200 K higher than for the same clusters on ceria.^26^ With this comprehensive picture of the fundamental dynamics of Pt_*n*_/Fe_3_O_4_(001) in UHV in mind, the question arises as to whether this highly dynamic system can survive near-ambient pressure conditions and elevated temperatures. To address this question, we have placed Pt_*n*_ clusters on Fe_3_O_4_(001) into more harsh environments in a NAP-XPS study at MAX IV, Lund.

**Fig. 4:**
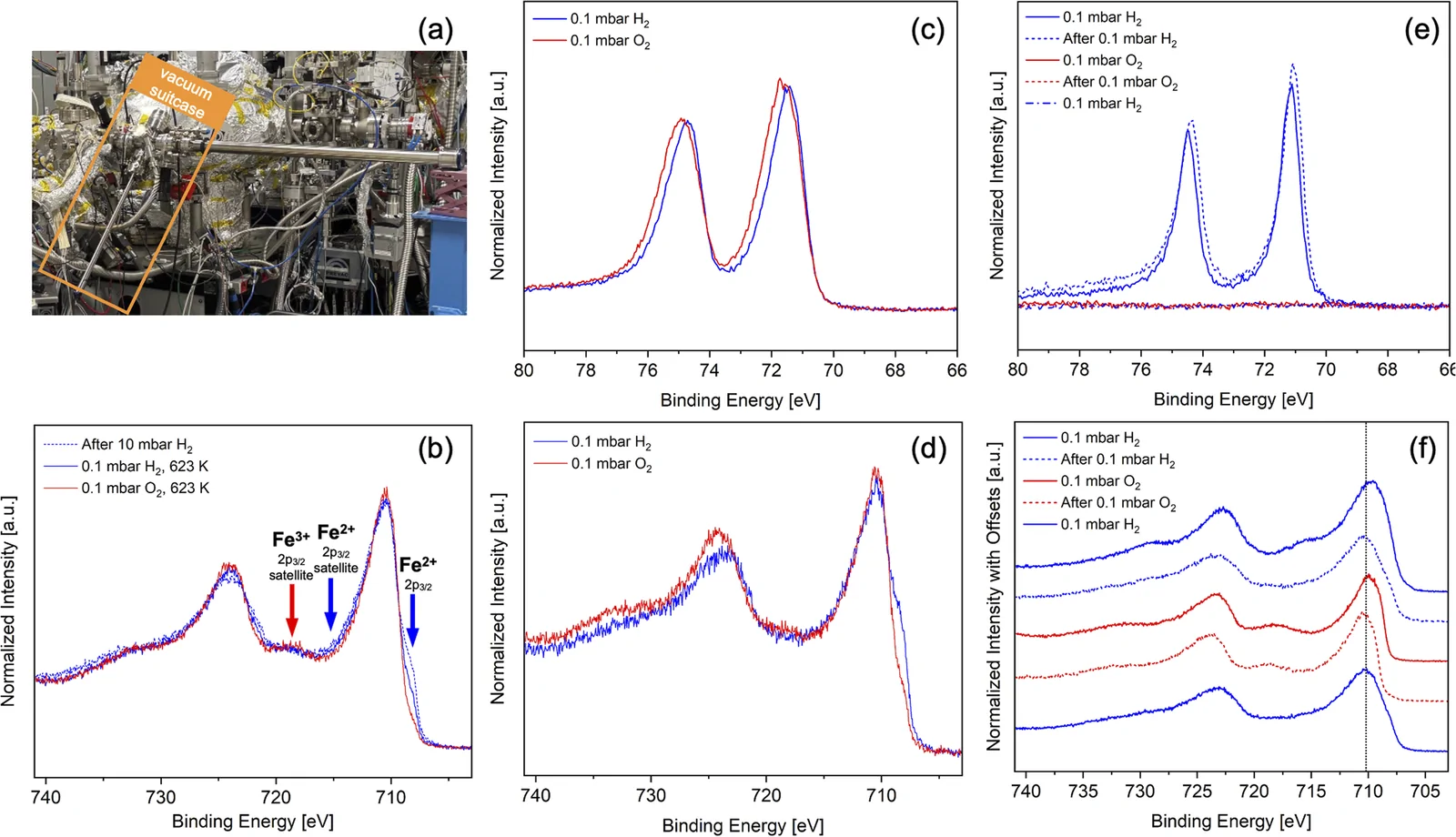
Evolution of Pt_10_ clusters on Fe_3_O_4_(001) under redox conditions from UHV to near-ambient pressures. (a) The vacuum suitcase in which the samples were transported (orange box). (b) The Fe 2p region bare of Fe_3_O_4_(001) after a treatment in 10 mbar H_2_ (dashed), and during exposure to 0.1 mbar H_2_ (blue) and 0.1 mbar O_2_ (red) at 623 K. (c) The Pt 4f region and (d) the Fe 2p region of Pt_10_/Fe_3_O_4_(001) in 0.1 mbar H_2_ (blue) and 0.1 mbar O_2_ at RT (red). (e) The Pt 4f region and (f) the Fe 2p region of Pt_10_/Fe_3_O_4_(001) during and in between redox treatments in 0.1 mbar H_2_ and 0.1 mbar O_2_ at 573 K, from top to bottom with individual vertical offsets.

Fig. 4 presents the evolution of Pt_10_/Fe_3_O_4_(001) in 0.1 mbar H_2_ and O_2_, respectively starting from RT and progressing to harsher redox conditions at elevated temperatures. It shows that near-ambient pressure and elevated temperature rapidly drive the system into states that are fundamentally different from those characterized in UHV. In contrast to Pt_*n*_/SiO_2_/Si, where the native silica layer and the underlying semiconductor remain structurally inert during transport, the Fe_3_O_4_(001) support is intrinsically much more dynamic. To minimize uncontrolled restructuring and contamination by ambient oxygen and water, these samples were transported to the beamline at MAX IV in a vacuum suitcase (shown in Fig. 4a) after their preparation in Munich and vacuum transferred to the measurement chamber.

We first consider mild redox conditions at RT on a Pt_10_/Fe_3_O_4_(001) sample after transport. Fig. 4c and d display the Pt 4f spectra and the corresponding Fe 2p spectra, respectively. Overall, the sample remains close to the well-characterized UHV state and shows only small changes. The Pt 4f peak intensity remains largely similar when switching from reducing to oxidizing conditions but shifts to slightly higher binding energies. Similar to the Pt_*n*_ clusters on n-SiO_2_, the shift is small enough that it likely arises from O adsorbates rather than true Pt oxidation. In the Fe 2p region, a low binding energy shoulder is visible in H_2_ and disappears in O_2_, which can be assigned to Fe^2+^ species.^38,70^ At the same time, the line shape between the peaks of the doublet changes slightly as well, in line with an Fe^2+^ satellite intensity change.^38,70^ We can thus conclude that the Pt_*n*_/Fe_3_O_4_(001) system is generally stable in 0.1 mbar gas environments at RT and only small redox changes occur in the support.

The bare Fe_3_O_4_(001) sample behaves similarly, even at elevated temperatures. Fig. 4b shows the Fe 2p spectra of magnetite after initial treatment in 10 mbar H_2_ at RT, and subsequent heating at 623 K in 0.1 mbar H_2_ (where signal intensities are superior to 10 mbar H_2_) and 0.1 mbar O_2_. Similar to the observation on the RT-treated cluster sample, reducing conditions lead to an Fe^2+^ shoulder and a satellite feature, which disappear in oxidizing conditions. These observations indicate that, in the absence of Pt, the Fe_3_O_4_ surface stoichiometry is comparatively stable under the temperatures, pressures and timescales used here.

The behavior changes drastically when we heat Pt_10_/Fe_3_O_4_(001) in H_2_ or O_2_ gas atmospheres. Fig. 4e and f again show the Pt 4f spectra and corresponding Fe 2p spectra. This Pt_10_/Fe_3_O_4_(001) sample was initially heated to 623 K in UHV, then exposed to 10 mbar H_2_ at RT, before performing the here-presented experiments at 573 K at 0.1 mbar pressure. According to our sintering study in UHV,^23,26^ Pt_10_ clusters on this support become encapsulated from about 523 K already, but start to sinter *via* Smoluchowski ripening only at or just beyond approximately 623 K. Hence, in a rough approximation (setting aside the influence of different annealing times), we are in a temperature regime of encapsulated, but still mostly size-selected clusters.

Fig. 4e shows a clear Pt 4f doublet after the initial H_2_ treatment, but as soon as the gas is switched to O_2_, no detectable Pt signal remains visible for either probing depth. We exclude encapsulation under an atomically thin oxide layer as an explanation. While the complete disappearance of the Pt signal could in principle be caused by Pt evaporation, the deep burial of Pt clusters in the magnetite support seems most plausible: in recent work on Pt/TiO_2_(110) – another support with high interstitial concentration – we could monitor the burial of Pt clusters and nanoparticles as passive bystanders during oxidative titania layer growth *in situ via* STM and XPS.^50^ A similar behavior thus seems likely in the present case. As on Pt/TiO_2_(110), returning to reducing conditions does not recover the Pt signal, further supporting the hypothesis of a deep burial rather than shallow encapsulation of the clusters. Such extensive overgrowth over metal particles on the single crystal surface is facilitated by its vast bulk, so even small interstitial densities in the bulk can amount to significant growth at the surface. In contrast, powder titania samples have a larger surface-to-bulk ratio, resulting in less extensive overgrowth. Nevertheless, a switching between two types of encapsulation films in H_2_ and O_2_, respectively, and their removal in a mixture of 60 mbar H_2_ and 700 mbar O_2_ has been reported on Pt/TiO_2_.^48,49^ Through a direct NAP-XPS comparison of Pt on titania powder and single crystalline supports, we could rationalize this contrast by the decisive role of the overall available number of interstitials.^50^

Returning to the experiments on Pt_*n*_ clusters on magnetite, we further find significant modification of the support during redox annealing (see Fig. 4f). The Fe^2+^ low binding energy shoulder and satellite features disappear in oxidizing conditions, leading to the pronounced appearance of the remaining Fe^3+^ satellite. After pumping out O_2_, the Fe 2p line shape remains largely unchanged, demonstrating that the oxidized state is (meta)stable in UHV on the experimental timescale. Only after subsequent reintroduction of H_2_ do the Fe^2+^ shoulder and satellite appear again; the reduction thus occurs spontaneously even when Pt is buried and thus no longer available at the surface. Moreover, the Fermi edge is lost upon oxidation but partially recovered upon subsequent exposure to 0.1 mbar H_2_ (see SI, Fig. S4), pointing to significant oxidation of the magnetite support. Indeed, the observed line shapes are comparable to those reported for hematite, Fe_2_O_3_.^70^ This stark contrast between the bare and Pt_10_-loaded magnetite samples suggests that the presence of Pt facilitates the formation of a strongly oxidized state of the surface, *e.g. via* enhanced oxygen activation and spillover, which then leads to Pt burial during reoxidation.

The combined picture that emerges from Fig. 4 is that Pt_*n*_ clusters on magnetite constitute a highly dynamic and fragile catalyst system under near-ambient pressure conditions. At RT, the Pt_*n*_ clusters reside in an electron-poor Pt^*δ*+^ state due to electronic metal–support interaction and magnetite remains in its mixed valence, semi-metallic state under 0.1 mbar redox conditions. Under harsher oxidizing conditions at elevated temperatures, however, the support oxidizes, losing its Fe^2+^ satellite, evolving towards a hematite-like surface, while Pt becomes deeply buried and spectroscopically invisible with the photon energies used here. Such burial renders the Pt clusters inaccessible for gas-phase reactants and thus effectively removes them as active catalytic sites, even though they may still persist structurally beneath the surface. Just like in the cases of Pt_*n*_ clusters on SiO_2_ and CeO_2_, the use of size-selected clusters ensures that we can distinguish between subtle changes like sintering onset and O adsorption/desorption.

These findings closely mirror our recent study on Pt particles on TiO_2_(110), where we distinguished between classical SMSI-induced encapsulation by reductive treatments and a non-classical deep burial mechanism in oxidizing conditions, driven by reoxidation of a highly reduced, Ti interstitial-rich support. There, we argued that the availability of mobile cation interstitials controls the extent and rate of overlayer growth, and that bulk single-crystalline supports provide a much larger defect reservoir than nano-sized powder grains. In the present magnetite system, which is intrinsically dominated by cation interstitials, the analogy is striking: Fe cation mobility enables rapid restructuring and the formation of a thick layer that can completely bury Pt_*n*_ clusters in O_2_ at 573 K. Whether such deeply buried Pt could be dynamically re-exposed under alternating redox conditions, as suggested for Pt/TiO_2_ powders by Willinger and co-workers,^48,49^ remains an open question; our current NAP-XPS experiments on single-crystals do not show any re-emergence of Pt after returning to H_2_, indicating that, at least under the conditions probed here, the burial is effectively irreversible.

## Conclusions

Our results highlight that atomically precise, size-selected clusters on well-defined oxide supports provide a powerful platform to disentangle the different dynamics governing heterogeneous catalysis. On the electronically tunable, but chemically inert SiO_2_/Si support, we demonstrate that the same Pt_*n*_ cluster size can be prepared in distinct charge states without changing the local cluster–support interface. As a result, cluster dimensionality changes from (hemi)spherical to 2D island-like. This controlled tuning allows us to study the role of cluster charge/dimensionality at comparable particle–support interfaces and the oxidation behavior, sinter resistance and response to ammonia oxidation mixtures with subtle changes in Lewis acidity. On CeO_2_(111), in contrast, the dynamics are driven by O vacancies on the support surface. Experimental control of O vacancy concentration again leads to a transition from 3D to 2D cluster geometries during sintering on reduced ceria, stabilized by oxygen vacancies. The Pt_*n*_/ceria system has been shown to provide a promising route to reversible cluster redispersion and catalyst self-repair *via* suitable redox protocols. We found that the proposed Pt^2+^ incorporation at step edges starts at the same temperature where Ostwald ripening sets in. On Fe_3_O_4_(001), finally, cation interstitials dominate the dynamics. Here, modest near-ambient pressures at RT leave Pt_*n*_ clusters and the magnetite support largely unchanged, but heating in 0.1 mbar O_2_ rapidly drives the interface into an oxidized, hematite-like overgrowth, resulting in deep burial of the Pt clusters that is not reversed by subsequent H_2_ treatment. We have discussed these findings in the context of another interstitial-driven, yet also O vacancy stabilizing support – Pt particles on TiO_2_(110) – which shows closely comparable results to Pt_*n*_/Fe_3_O_4_.

Taken together, these case studies delineate a range of Pt_*n*_ cluster evolution possibilities in such dynamically evolving active sites: from electronically tuned, but structurally robust clusters on non-reducible oxides, through vacancy-mediated reshaping and self-repair on ceria, to interstitial-mediated encapsulation and deep burial on magnetite and titania. These findings suggest that model catalyst dynamics must be engineered within a suitable window: sufficient to enable redispersion, restructuring and charge-state modulation under reaction conditions, yet limited enough to avoid catastrophic burial of the active metal. On the experimental level, this means that support defect chemistry (vacancies *vs.* interstitials, defect density and mobility), cluster dimensionality, and charge state can be controlled as independent design parameters *via* support stoichiometry and morphology, as well as cluster generation and catalyst activation. The size-selected cluster approach, combined with transport strategies for synchrotron-based methodologies, provides the experimental precision needed to track how precursor structures are converted into active sites. The systems presented here form a basis for deliberately coupling O vacancy and interstitial dynamics, cluster fluxionality and potentially even external stimuli in order to design catalysts that exploit structural and electronic dynamics as an intrinsic element of long-term activity, selectivity and self-repair.

## Author contributions

LJF: data curation, writing – review & editing; MH: investigation, methodology, data curation, formal analysis, visualization, writing – review & editing; JR: investigation, data curation, formal analysis, visualization, writing – review & editing; MKri: investigation, methodology, software, writing – review & editing; SK: investigation, data curation, formal analysis, writing – review & editing; MD: investigation, writing – review & editing; MDR: investigation, writing – review & editing; MKra: investigation, writing – review & editing; AS: investigation; SZ: investigation; UH: resources, writing – review & editing; HB: investigation, resources, supervision, writing – review & editing; FE: investigation, methodology, data curation, formal analysis, visualization, funding acquisition, writing – review & editing; BAJL: investigation, visualization, methodology, supervision, project administration, funding acquisition, writing – original draft.

## Conflicts of interest

There are no conflicts to declare.

## Supplementary Material

FD-267-D6FD00002A-s001

## Data Availability

Data for this article are available at Zenodo at https://doi.org/10.5281/zenodo.18173637. Supplementary information (SI) is available. See DOI: https://doi.org/10.1039/d6fd00002a.
