# Supplementary material for: Evolution of size-selected Pt cluster catalysts on prototypical oxide supports
Source: Faraday Discuss. 2026 Mar 11. Online ahead of print. doi: 10.1039/d6fd00002a (PMC13196843; doi:10.1039/d6fd00002a)
Supplement: FD-OLF-D6FD00002A-s001 [file FD-OLF-D6FD00002A-s001.pdf]

Supplementary Information (SI) for Faraday Discussions.
This journal is © The Royal Society of Chemistry 2026

Faraday Discussions

Supporting Information

Evolution of size-selected Pt cluster catalysts on prototypical oxide supports

Lorenz J. Falling,[a] Maximilian Huber,[a,b] Johanna Reich,[a] Matthias Krinninger,[a] Sebastian Kaiser,[c] Markus Döblinger,[d] Marian D. Rötzer,[c] Maximilian Krause,[c] Andrey Shavorskiy,[e] Suyun Zhu,[e] Ueli Heiz,[c] Hendrik Bluhm,[b,‡] Friedrich Esch,[c] and Barbara A. J. Lechner* [a,f]

[a] Functional Nanomaterials Group & Catalysis Research Center, Department of Chemistry, TUM School of Natural Sciences, Technical University of Munich, Lichtenbergstr. 4, 85748 Garching, Germany

[b] Chemical Sciences Division & Advanced Light Source, Lawrence Berkeley National Laboratory, Berkeley, California 94720, United States

[c] Chair of Physical Chemistry & Catalysis Research Center, Department of Chemistry, TUM School of Natural Sciences, Technical University of Munich, Lichtenbergstr. 4, 85748 Garching, Germany

[d] Department of Chemistry & Center for NanoScience (CeNS), University of Munich (LMU), Butenandtstr. 11, 81377 Munich, Germany

[e] MAX IV Laboratory, Lund University, Lund 221 00, Sweden

[f] Institute for Advanced Study, Technical University of Munich, Lichtenbergstr. 4, 85748 Garching, Germany

‡ present address: Fritz Haber Institute of the Max Planck Society, Faradayweg 4-6, 14195 Berlin, Germany

Survey scans of a representative SiO_2/Si sample

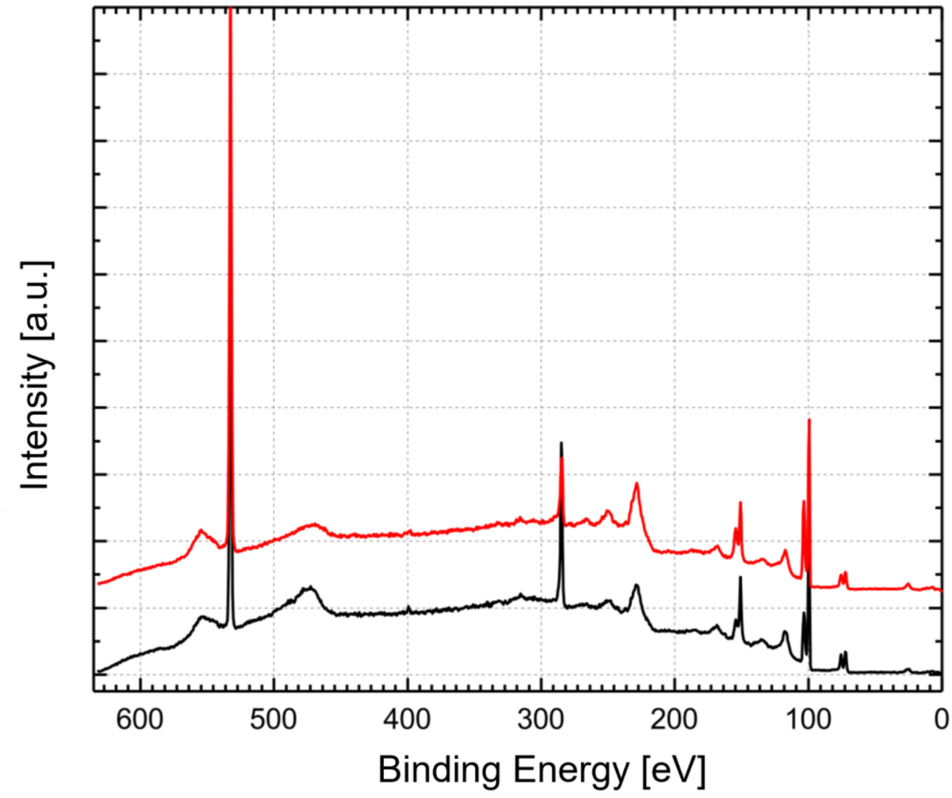

Figure S1. Survey scans of a representative Pt_{20}/SiO_2/n-Si sample as introduced into the NAP-XPS after transport (black) and after some oxidative treatment (red), recorded with a photon energy of 735 eV. For clarity, the scans are shown offset.

Pt distribution on suspended and Si-supported regions of a Si_3N_4 TEM grid

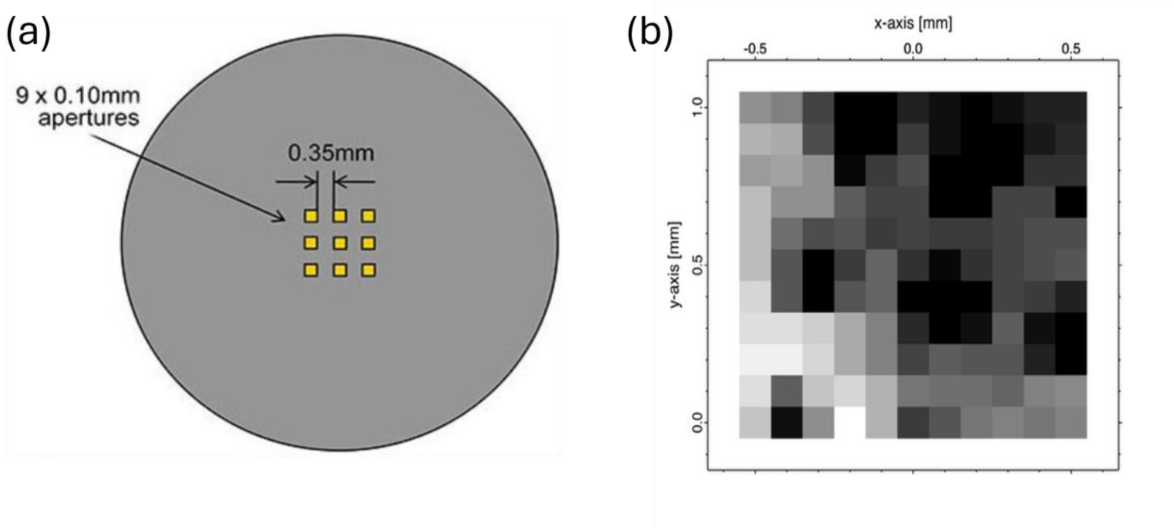

Figure S2. Pt distribution on a Si_3N_4 TEM grid. (a) Layout of the nine apertures in the PELCO silicon nitride TEM grid by TedPella, covered with an 8 nm thick Si_3N_4 film [Image from http://www.tedpella.com/grids_html/silicon-nitride-details.htm, 01-Feb-2017]. (b) The maximum signal of the Pt 4f peak was recorded at different points, moving in 0.1 mm steps in the 1 x 1 mm^2 central region of the TEM grid, upon which a coverage of 1 atom/nm^2 of Pt_8 clusters was deposited. To rule out background-induced effects, several scans across the peaks in the Pt 4f region were also recorded (not shown), indicating that the background level was comparable throughout the measurement region. Darker colors in the 2D plot indicate lower Pt signal, lighter gray/white colors indicate higher intensity. Eight of the nine holes in a tilted square arrangement are clearly visible as dark regions, i.e. areas with a reduced amount of Pt. This Pt distribution results from charging of the suspended Si_3N_4 membrane areas during Pt_n^+ cluster deposition, leading to a deflection of the incoming positively charged clusters, while the charges can be neutralized effectively on those areas of the Si_3N_4 film supported by bulk Si, where the higher coverage is found. The HAADF-STEM images shown in the main manuscript were recorded in the regions of suspended nitride, while all XPS data is from supported areas.

Fe 3p XPS energy calibration

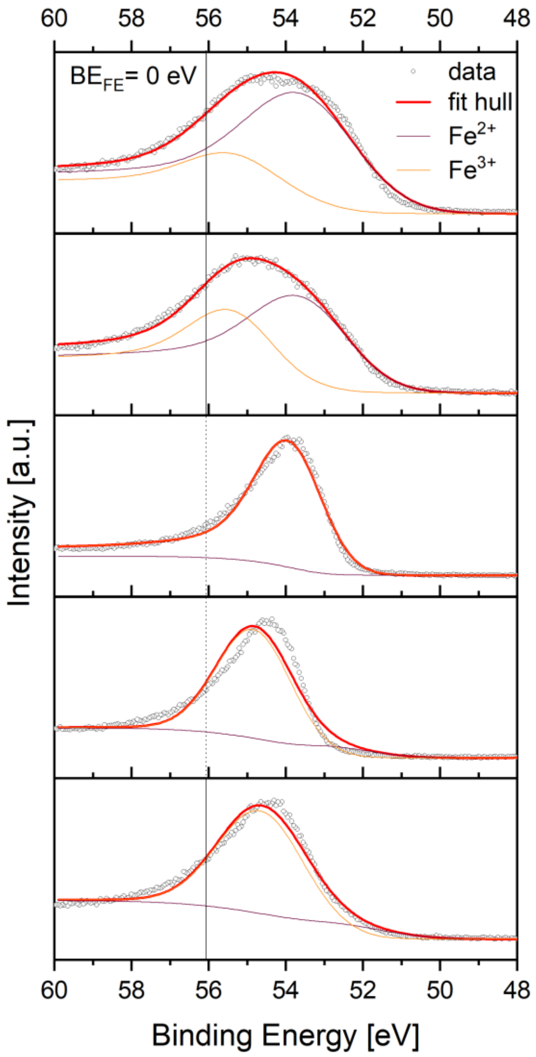

Figure S3. Uncalibrated Fe 3p spectra of $Pt_{10}/Fe_3O_4(001)$ at 573 K during (top panel) and after 0.1 mbar H_2 (second panel), during (third panel) and after 0.1 mbar O_2 (fourth panel), and in 0.1 mbar H_2 after all treatments (bottom panel), corresponding to the measurements shown in Fig. 4e-f in the main manuscript. The spectra are fitted with two Doniach-Šunjić functions with an asymmetry parameter fixed at 0.15, and a distance between the two components fixed at 1.8 eV. The width of the Fe^{3+} feature is fixed at 0.84 times the width of the Fe^{2+} function, to best represent all spectra. In contrast to the Fermi edge, which disappears in oxidative conditions (see Fig. S4), the Fe^{3+} peak is always present and can thus be used for binding energy referencing. We shift all spectra such that the peak of the orange component is at 56.05 eV (vertical line, dashed where no Fermi edge could be measured), putting the Fermi edge position at 0.0 eV on average.

Fermi edge of $Pt_{10}/Fe_3O_4(001)$ in redox conditions

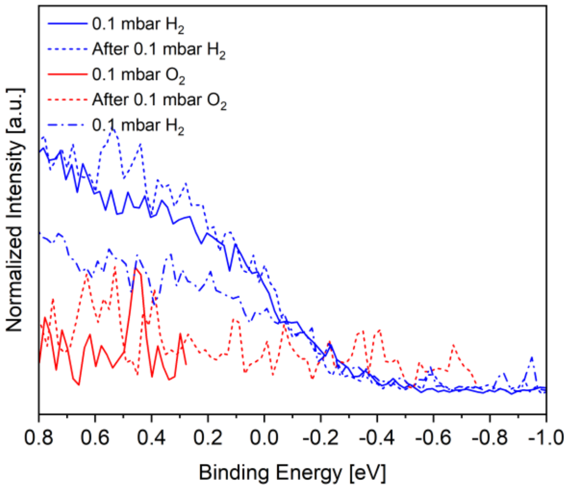

Figure S4. Fe 3p calibrated Fermi edges of $Pt_{10}/Fe_3O_4(001)$ at 573 K during and in between redox treatments in 0.1 mbar H_2 and 0.1 mbar O_2 at 573 K. Oxidative conditions eliminate the Fermi edge and the highest lying states are shifted to more positive binding energies.

Thickness of the silica film on SiO_2/Si during oxidative treatment

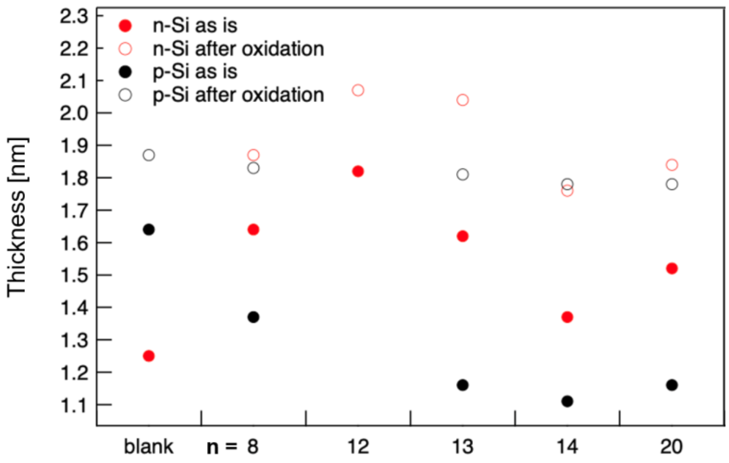

Figure S5. Thickness of the SiO_2 layer on the examined samples before (filled circles) and after (open circles) the cleaning procedure, both on n-type (red) and p-type (black) Si substrates. The oxide thickness t_{ox} was calculated by $t_{ox} = \lambda_{ox} \ln [1+(I_{ox} \lambda_M N_M)/(I_M \lambda_{ox} N_{ox})]$; with I_x, λ_x, and N_x being the intensity, inelastic mean free path, and atomic density of the metal (M) or oxide (ox), respectively.

Response of Pt 4f of Pt_n/SiO_2/n-Si to various gas atmospheres

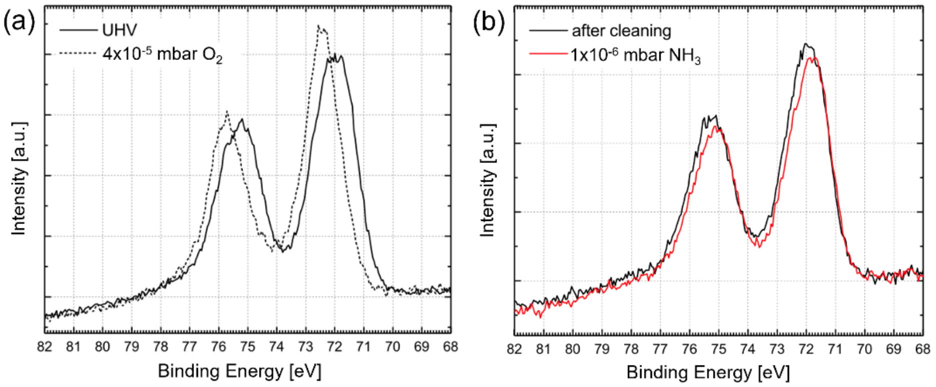

Figure S6. (a) Pt 4f spectra of Pt_{40}/SiO_2/n-Si in different gas atmospheres at RT: (a) as introduced into UHV (solid line) and in 4 x 10^{-5} mbar O_2 (dashed line), and (b) after cleaning (black) and during dosing of 1 x 10^{-6} mbar NH_3 (red), following previous cleaning in O_2, exposure to the reaction mixture at RT and heating heating to 423 K in NH_3.

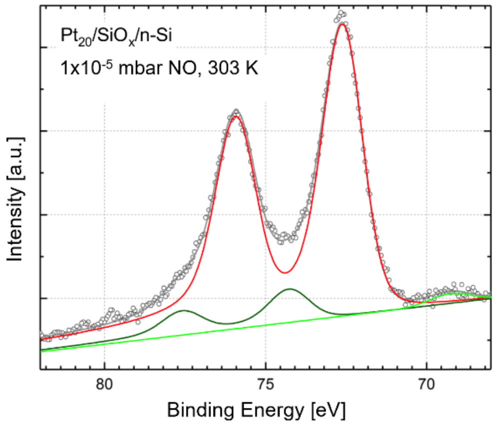

Figure S7. Pt 4f spectrum of Pt_{20}/SiO_2/n-Si in 1 x 10^{-5} mbar NO at 303 K on a sample that was previously exposed to reaction atmosphere and heated to 423 K in NO, including a fit with two components.

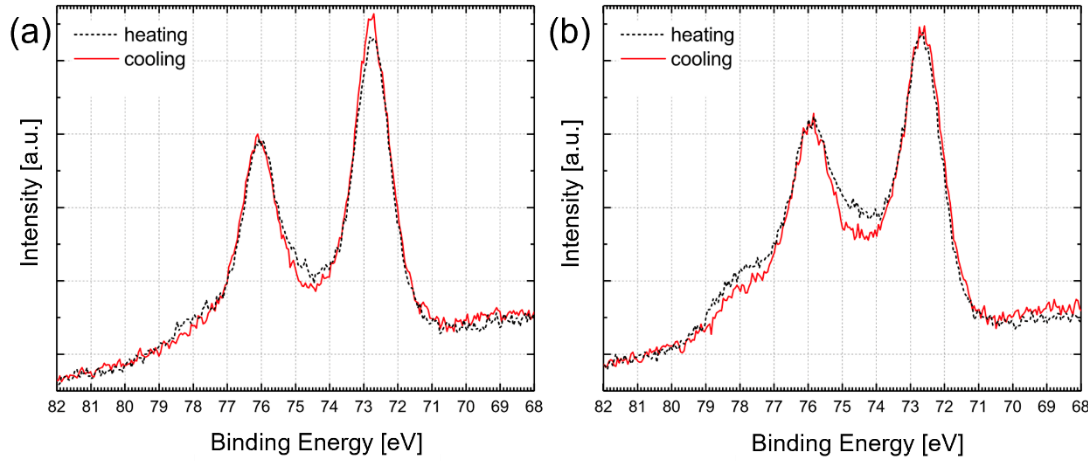

Figure S8. Comparison of the Pt 4f spectrum of (a) Pt_{20}/SiO_2/p-Si and (b) Pt_{20}/SiO_2/n-Si after cleaning and in ammonia oxidation conditions, i.e. 4 x 10^{-5} mbar O_2 and 1 x 10^{-5} mbar NH_3 at 303 K. The black dashed curves were recorded at 303 K during a heating ramp up to 423 K and the red curves at 303 K during the cooling ramp back down to RT. The oxidized species discussed in the main manuscript are clearly visible on the n-type sample and only form to a much smaller degree during the heating ramp on the p-type sample (not at all during the cool-down).
